# Supplementary material for: Bacterial volatile organic compound specialists in the phycosphere
Source: ISME J. 2025 Oct 15;19(1):wraf229. doi: 10.1093/ismejo/wraf229 (PMC12599314; doi:10.1093/ismejo/wraf229)
Supplement: Supplementary_Materials_wraf229 [file supplementary_materials_wraf229.docx]

Supplementary Materials for

**Bacterial volatile organic compound specialists in the phycosphere**

Vaishnavi G. Padaki, Xavier Mayali, Peter K. Weber, Stephen J. Giovannoni, Kaylene Abraham, Kerry Jacobs, Lindsay Collart, Kimberly H. Halsey^*^

^*^Corresponding author. Email: [halseyk@oregonstate.edu](mailto:halseyk@oregonstate.edu)

**This file includes:**

Supplemental information on sample preparation for nanoSIMS analysis

Supplemental information on isotopic analysis by nanoSIMS

Figs. S1 to S4

**Other supplementary materials for this manuscript include the following:**

Tables S1 to S7

Supplementary Table 1: Estimated bacterial cell counts for *Marinobacter* and *Rosiebium*

Supplementary Table 2: Genes encoding volatile organic compound oxidizing proteins in *Marinobacter*, *Roseibium*, *Stappia*, *Rhodophyticoloa*, and *Yoonia*

Supplementary Table 3: Volatile organic compounds detected in exponential and stationary phases of *P. tricornutum*

Supplementary Table 4: Volatile organic compounds depleted in *P. tricornutum*-bacteria cocultures in exponential phase

Supplementary Table 5: Volatile organic compounds not depleted in *P. tricornutum*-bacteria cocultures

Supplementary Table 6: Cell properties in axenic *P. tricornutum* cultures and in *P. tricornutum*-bacteria cocultures

Supplementary Table 7: Volatile organic compounds depleted in *P. tricornutum*-bacteria cocultures in stationary phase

**Supplementary information:**

*NanoSIMS sample preparation*:

Samples (5 - 10 ml) were immediately fixed with 10% formalin, incubated for 30 min at 4o C, filtered onto 0.2 μm polycarbonate filters, and washed three times with nanopore Milli-Q water. To account for non-specific 13C binding, killed-controls were grown with no benzene, fixed with formaldehyde, incubated with 13C benzene for 30 min at 4o C, filtered, and washed. All filters were air-dried and then cut into small wedges, adhered to aluminum disks using conductive tabs (#16084-6, Ted Pella, Redding, CA), and shipped at ambient temperature to Lawrence Livermore National Laboratory. PT-*Rhodophyticola* required hydrofluoric acid cleaning (1% HF for 10 min followed by rinse in Milli-Q water) due to a coating of particulate matter blocking cells.

*Brief explanation of isotopic measurement using nanoSIMS*:

Samples were sputter-coated with ~5 nm of gold. Analysis locations were first sputtered to a depth of ~60 nm with the primary 133Cs+ ion beam set to ~100 pA before analysis with 2 pA (150 nm beam diameter at 16 keV). Rastering was performed over 20 x 20 μm analysis areas with a dwell time of 1 ms pixel-1 for 19-30 scans (cycles) and generated images containing 256 x 256 pixels, with sputtering equilibrium to a depth of ~60 nm. After tuning the secondary ion mass spectrometer for mass resolving power of ~7000 (1.5x corrected), secondary electron images and quantitative secondary ion images were simultaneously collected for 12C2-, 13C12C-, 12C14N-, and 12C15N- on individual electron multipliers in pulse counting mode. All NanoSIMS datasets were initially processed using L’Image (http://limagesoftware.net) to perform dead time and image shift correction of ion image data before creating 13C/12C and 15N/14N ratio images. To quantify substrate incorporation, cells were identified based on 12C14N- images, and regions of interest (ROIs) were drawn manually around each bacterial cell. In order to calculate how much C or N a cell incorporated from a substrate that was isotopically labeled, we calculated Xnet, either Cnet or Nnet (Dekas et al., 2019; Pett-Ridge et al., 2022), which represent the fraction of a cell’s biomass that originated from the added isotope labeled substrate (here, 13C-benzene and 15N-leucine for Cnet and Nnet, respectively) and used the killed control ($f_{X_{i}}$ ), the measurement at the final

timepoint ($f_{X_{f}})$, and the isotope fraction of the substrate ($f_{X_{s}}$), which assumed negligible unlabeled benzene (12C-benzene):

$$X_{net\%}= \frac{f_{X_{f}}-f_{X_{i}}}{f_{X_{s}}-f_{X_{i}}} \times100\%$$

**
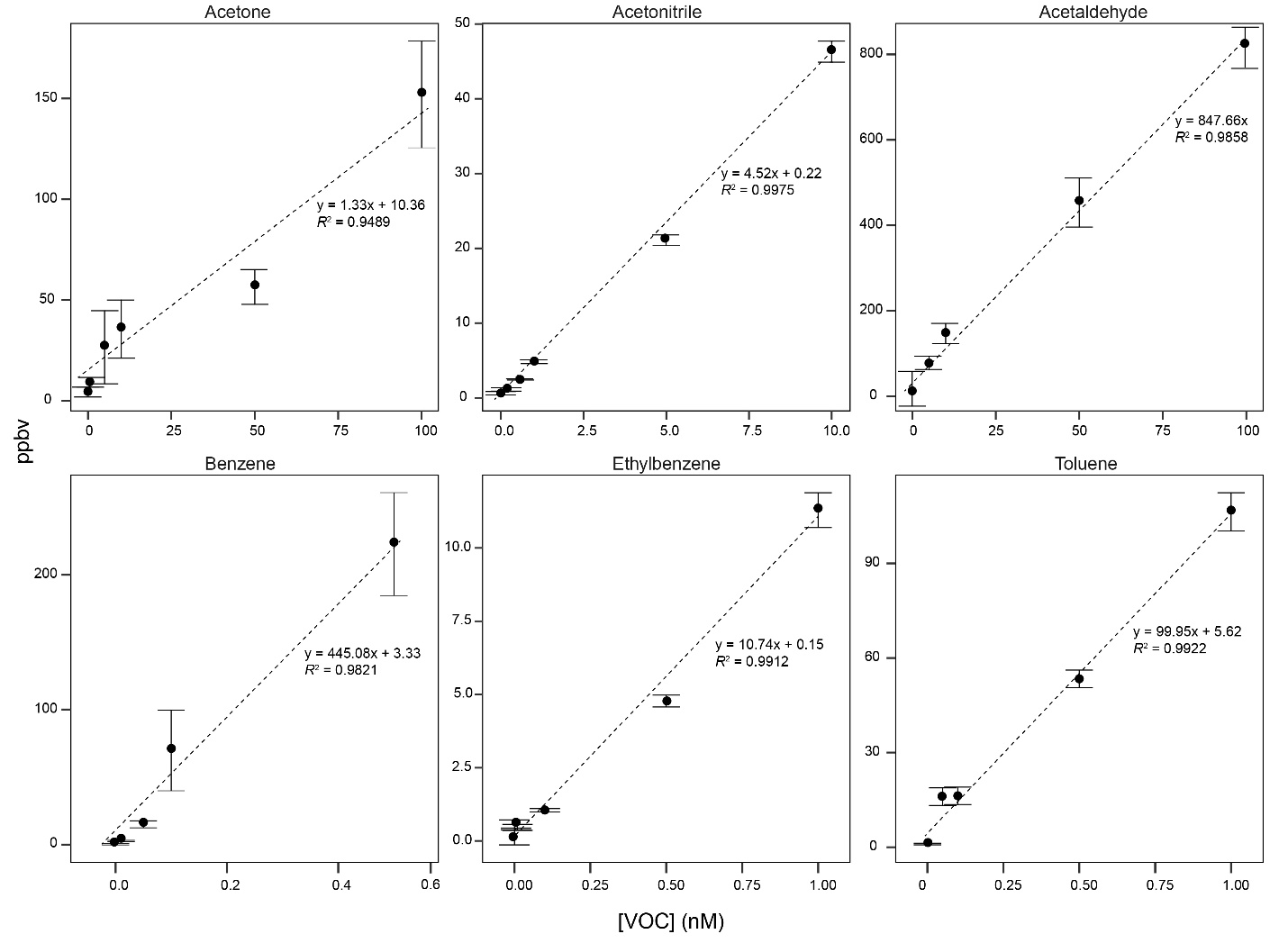
**

**Supplementary Fig. S1.** Dilution series of standard VOCs. Each sample point indicates ppbv values detected at the known VOC concentration. Error bars give standard error of triplicate independent measurements.


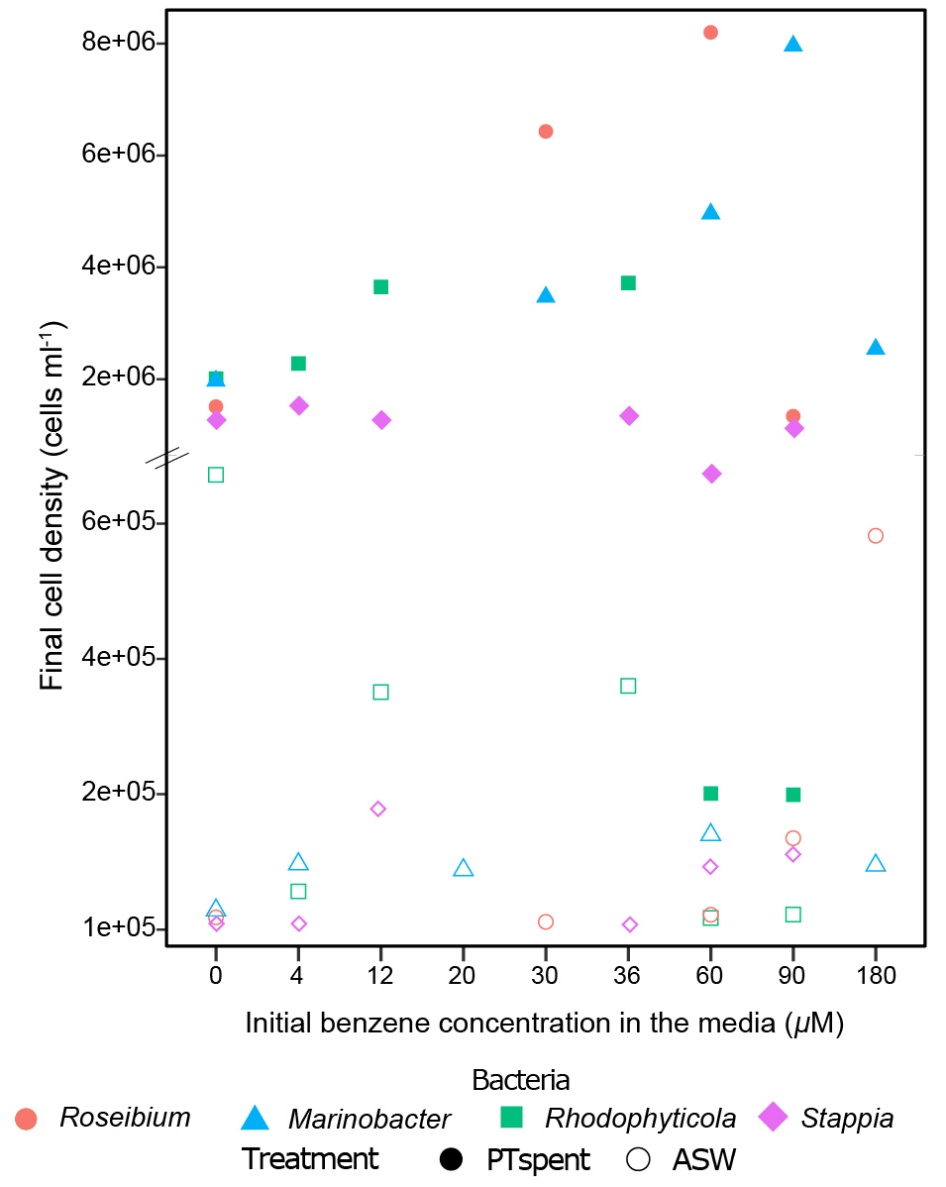


**Supplementary Fig. S2.** Final cell densities measured in benzene dose experiments. Each bacterium was inoculated into PTspent (filled symbols) and ASW media (open symbols) with initial benzene concentrations 0-180 µM. *Roseibium* (circles), *Marinobacter* (triangles), and *Rhodophyticola* (squares).


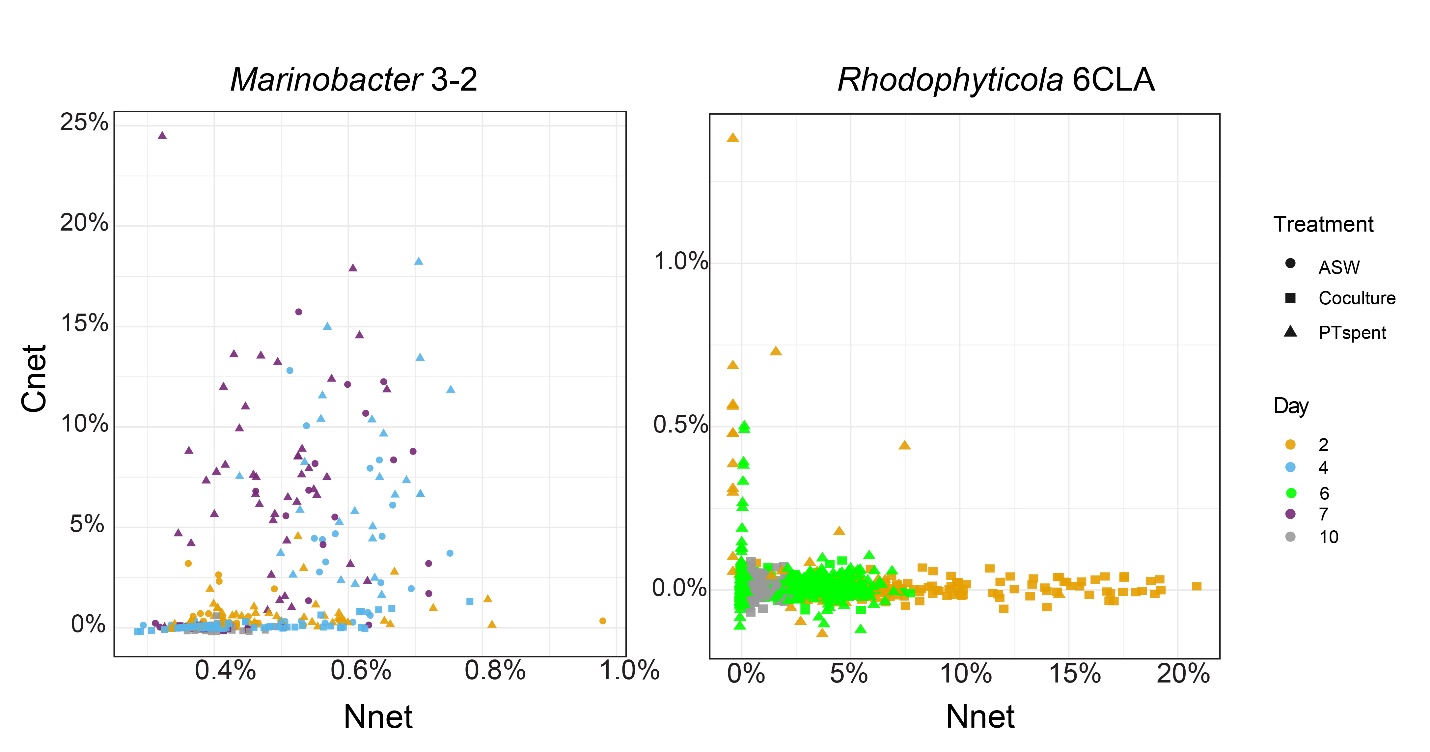


**Supplementary Fig. S3.** C_net_ vs N_net_ in *Marinobacter* and *Rhodophyticola*. Percent of bacterial biomass derived from ^13^C-benzene (Cnet) or ^15^N-leucine (Nnet) following incubation for two to ten days. C_net_ and N_net_ in killed cell controls were subtracted from C_net_ and N_net_ in growing cells.

**
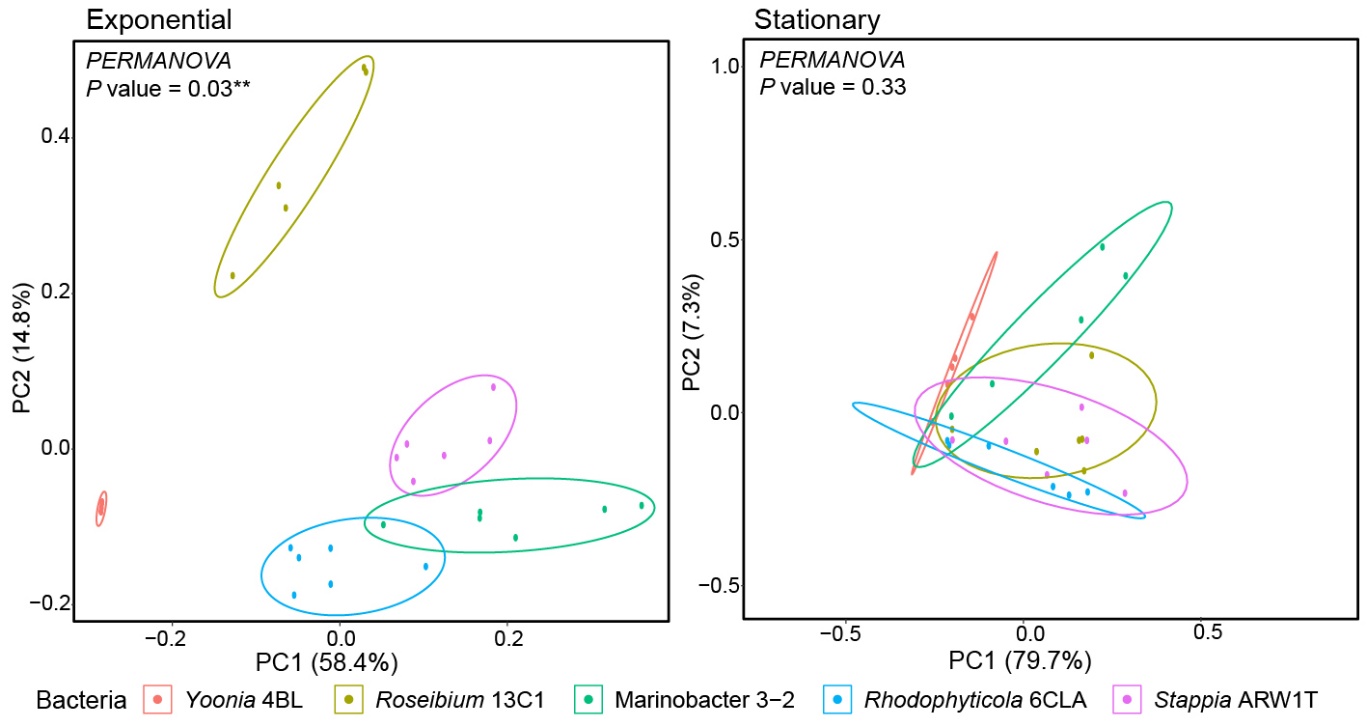
**

**Supplementary Fig. S4.** Principal coordinate analysis of depleted VOCs in *P. tricornutum*-bacteria cocultures in exponential (left) and stationary (right) growth phases.
